# Bacterial infections and cancer

**DOI:** 10.15252/embr.201846632

**Published:** 2018-10-22

**Authors:** Daphne van Elsland, Jacques Neefjes

**Affiliations:** ^1^ Oncode Institute and Department of Cell and Chemical Biology Leiden University Medical Center LUMC Leiden The Netherlands

**Keywords:** bacteria, cancer, effectors, infection, signalling, Cancer, Microbiology, Virology & Host Pathogen Interaction, Signal Transduction

## Abstract

Infections are estimated to contribute to 20% of all human tumours. These are mainly caused by viruses, which explains why a direct bacterial contribution to cancer formation has been largely ignored. While epidemiological data link bacterial infections to particular cancers, tumour formation is generally assumed to be solely caused by the ensuing inflammation responses. Yet, many bacteria directly manipulate their host cell in various phases of their infection cycle. Such manipulations can affect host cell integrity and can contribute to cancer formation. We here describe how bacterial surface moieties, bacterial protein toxins and bacterial effector proteins can induce host cell DNA damage, and thereby can interfere with essential host cell signalling pathways involved in cell proliferation, apoptosis, differentiation and immune signalling.

**Glossary**
Apc: Adenomatous polyposis coli
BFT: *Bacteroides fragilis* toxin
CagA: Cytotoxin‐associated gene A
CCL5: Chemokine (C‐C motif) ligand 5
CDK1: Cyclin‐dependent kinase 1
CDT: Cytolethal distending toxin
DDR: DNA damage responses
DSBs: Double‐strand DNA breaks
EF‐2: Elongation factor 2
EGFR: Epidermal growth factor receptor
ER: Endoplasmic reticulum
ERK: Extracellular signal‐regulated kinase
FadA: Fusobacterium adhesion A
FCP: *Francisella‐*containing phagosome
IKK: Iκβ kinase
IL: Interleukin
JNK: C‐Jun N‐terminal kinase
LPS: Lipopolysaccharides
MALT: Mucosa‐associated lymphoid tissue
MAPK: Mitogen‐activated protein kinase
MEK: Mitogen‐activated protein kinase kinase
MyD88: Myeloid differentiation primary response 88
NET1: Neuroepithelial cell‐transforming gene 1 protein
NF‐κβ: Nuclear factor‐κβ
OipA: Outer inflammatory protein A
PAK: p21‑activated kinase
Pks: Polyketide synthetase
Raf: Rapidly Accelerated Fibrosarcoma
SCV: *Salmonella*‐containing Vacuole
Tcf: T‐cell factor
TLR: Toll‐like receptor
VacA: Vacuolating cytotoxin A

## Introduction

Cancer development is the result of a series of genetic modifications that alter the normal control of cell growth and survival. These genetic alterations can be induced by a wide variety of external factors 1, including smoking, alcohol 2 and sunlight 3, 4. At least 75% of the head and neck cancers are caused by tobacco and alcohol 5 and 65–86% of the skin cancer risk can be attributed to sun exposure 4. In addition to these external factors, viral genomes have been retrieved from a variety of tumour samples 6 and this link has been further substantiated by many epidemiological studies (Table 1). For example, viral infections such as human papillomavirus and hepatitis B virus and hepatitis C virus have been associated with ~90% of cervical cancer cases 7 and ~80% of hepatocellular carcinoma cases 8, respectively.

**Table 1:** Epidemiological and experimental evidence for a link between microbial infections and cancer

Infectious agent	Type of micro‐organism	Cancer type
Epstein–Barr virus	Virus	Nasopharyngeal carcinoma, Burkitt lymphoma, immune suppression‐related non‐Hodgkin lymphoma, Hodgkin lymphoma, extranodal natural killer/T‐cell lymphoma (nasal type) 102
Hepatitis B virus	Virus	Hepatocellular carcinoma 102
Hepatitis C virus	Virus	Hepatocellular carcinoma, non‐Hodgkin lymphoma 102
Kaposi sarcoma herpesvirus	Virus	Kaposi sarcoma, primary effusion lymphoma 102
Human immunodeficiency virus 1	Virus	Kaposi sarcoma, non‐Hodgkin lymphoma, Hodgkin lymphoma, carcinoma of the cervix, anus, conjunctiva 102
Human papillomavirus type 16	Virus	Carcinoma of the cervix, vulva, vagina, penis, anus, oral cavity, and oropharynx and tonsil 102
Human T‐cell lymphotropic virus type 1	Virus	Adult T‐cell leukaemia and lymphoma 102
Merkel cell polyomavirus	Virus	Merkel cell carcinoma 103
*Opisthorchis viverrini*	Trematode	Cholangiocarcinoma 102
*Clonorchis sinensis*	Helminth	Cholangiocarcinoma 102
*Schistosoma heamatobium*	Trematode	Urinary bladder cancer 102
*Helicobacter pylori*	Bacterium	Non‐cardia gastric carcinoma, low‐grade B‐cell MALT gastric lymphoma 102
Alfatoxin (B1)	Mould (Aspergillus flavus)	Liver cancer 102
*Salmonella* Typhi	Bacterium	Gallbladder carcinoma 13
*Salmonella* Enteritidis	Bacterium	Colon carcinoma in the ascending and transverse parts of the colon 14
*Chlamydia trachomatis*	Bacterium	Carcinoma of the cervix and ovaries 104, 105

An even more compelling case for the link between viral infections and cancer arose from experiments showing that viruses exploit the host cell niche for their infection cycle and as a result stimulate mammalian growth‐inducing genes, leaving the cells in a cancerous state of uncontrolled cell division. It is now understood how viruses such as hepatitis B virus and human papillomavirus types 5 and 8 cause cellular transformation by inducing genetic instability through viral integration and through the activation of a large number of signalling pathways and cellular genes involved in oncogenesis, proliferation, inflammation and immune responses 9, 10.

Viruses do, however, represent only one segment of the microbiome that exploits the mammalian host during its infection cycle. Pathogenic moulds, helminths and bacteria intensively interact with mammalian host cells to ensure their survival. Although these microorganisms usually do not leave a genetically recognizable trait or piggyback on mammalian genes, such as illustrated by viral infections, strong epidemiological links exist between various microbiological infections and cancers (Table 1). Examples include connections between *Schistosoma haematobium* infections and bladder cancer 11, *Helicobacter pylori* (*H. pylori*) infections and gastric cancer 12, chronic *Salmonella* Typhi (*S*. Typhi) infections and gallbladder carcinoma 13, and *Salmonella* Enteritidis (*S*. Enteritidis) infections and colon carcinoma 14. Moreover, studies in germ‐free and antibiotic‐treated animals have indicated cancer‐promoting effects of microbiota in various experimental systems, varying from gastric 15, 16, colon 17, 18 and liver 19 cancers.

However, since microbiome–host interactions are extremely diverse, their exact contributions to cancer development are hard to pinpoint. Especially, pathogenic bacteria have been shown to manipulate and exploit the human host cell niche in various ways throughout various stages of their infection cycle. In this review, we will discuss how bacterial surface moieties, bacterial protein toxins and bacterial effector proteins interact with host cells, and how such encounters can result in the modification of essential host cell signalling pathways involved in cancer formation.

## Bacterial cell‐surface components and cancer development

The bacterial outer surface directly contacts host cells and consists of complex structures that include various antigenic moieties that activate host innate and adaptive immune responses. As a consequence, pathogenic bacteria have evolved a wide variety of outer‐surface modifications that ensure immune escape to afford significant survival opportunities. To abolish immune recognition and clearance, Gram‐negative bacteria cover their complex outer‐surface macromolecules with a polysaccharide‐rich capsule. These capsules limit complement activation by shielding deeper structures on the membranes of pathogenic variants of *Escherichia coli* (*E. coli*), *Streptococcus pneumoniae*,* Haemophilus influenzae* type b, *Neisseria meningitidis* and others, and prevent engulfment by professional phagocytes 20, 21, 22, 23. Unencapsulated mutants of these bacteria rarely cause an invasive infection and are highly attenuated in various infection models due to better opsonophagocytic clearance 22, 24, 25.

In addition to their shielding capsules, many bacterial pathogens have modified their surface‐exposed molecules, including lipopolysaccharides (LPS), flagella and peptidoglycans, to limit immune recognition. For example, *H. pylori* has LPS surface molecules that harbour “underacylated” lipid A molecules that are a poor substrate for host Toll‐like receptor (TLR)4 and as such evade innate immune sensing 26, 27. *Helicobacter pylori* also produces modified flagellin molecules that are not recognized by TLR5 to prevent TLR5‐mediated interleukin (IL)‐8 secretion and subsequent immune signalling 28. *Salmonella typhimurium* (*S. typhimurium*) expresses lipid A deacetylase PagL and a lipid A palmitoyltransferase PagP to modify lipid A, resulting in a 100‐fold decrease in lipid A‐mediated TLR4 activation and nuclear factor‐κβ (NF‐κβ) activation 29. These examples illustrate how bacterial pathogens modify their outer surface to escape immune recognition.

Pathogenic bacteria that favour an intracellular lifestyle express surface proteins that promote both host cell attachment and internalization. For example, pathogenic species of the *Neisseria* family express a variety of surface adhesins that mediate selective interaction with certain cell types, thereby allowing the exploitation of specialized host cell niches 30. In a similar fashion, fibronectin‐binding proteins of *Staphylococcus aureus* and *Borrelia burgdorferi* mediate the interaction between bacterium and host cell through the formation of tandem β‐zippers that stimulate bacterial engulfment by non‐phagocytic cells 31, 32.

**Figure 1:**
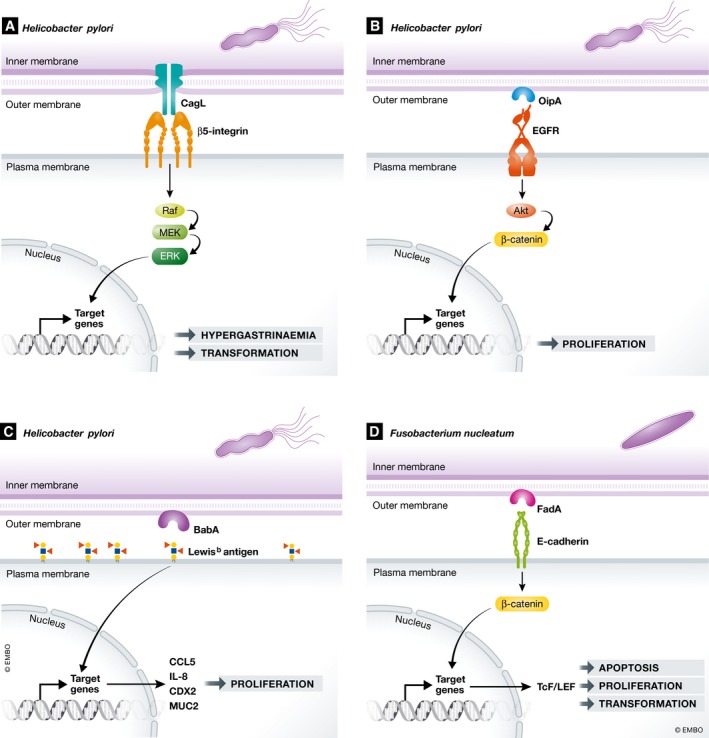
Bacterial outer‐surface components that manipulate host cell signalling cascades involved in cellular malignancy (A) *Helicobacter pylori* CagL binds β5‐integrin and induces downstream signalling of Raf, MEK and ERK pathways that play a central role in *H. pylori*‐induced gastrin production and cellular transformation. (B) *H. pylori* OipA activates EGFR and stimulates Akt and β‐catenin signalling, causing cell proliferation. (C) *H*. *pylori* BabA binds human Lewis(b) surface epitopes which increases levels of CCL5, IL‐8, CDX2 and MUC2, causing cell proliferation. (D) *Fusobacterium nucleatum* FadA binds to E‐cadherin, which releases β‐catenin that activates transcription factor Tcf/LEF which controls the transcription of genes involved in apoptosis, cell proliferation and transformation.

In general, these surface‐mediated assault strategies are aimed at facilitating bacterial survival within the host through both immune evasion and host invasion. However, to further control the host cell machinery, bacterial surface molecules also manipulate host cell signalling cascades and affect host cell integrity, which can coincidentally induce cellular malignancies. CagL is a type IV pilus adhesin of *H. pylori* that ensures the adherence of *H. pylori* to gastric epithelial cells and then controls a signalling cascade that induces upregulation of gastrin secretion. This results in hypergastrinemia, a major risk factor for the development of gastric adenocarcinoma. CagL binds β5‐integrin thus manipulating integrin‐linked kinase complexes and the downstream rapidly accelerated fibrosarcoma (Raf) kinase, the mitogen‐activated protein kinase kinase (MEK) and the extracellular signal‐regulated kinase (ERK) pathways (Fig 1A) 33. The outer inflammatory protein A (OipA) of *H. pylori* activates EGFR (epidermal growth factor receptor) and stimulates Akt and β‐catenin signalling, a phenotype observed in a number of different cancers, including gastric cancer (Fig 1B) 34, 35. OipA inactivation decreases β‐catenin nuclear localization *in vitro* and reduces the incidence of cancer in animal models 36. In addition, the blood group antigen‐binding adhesin BabA of *H. pylori* can bind human Lewis(b) surface epitopes which indirectly increases mRNA levels of proinflammatory cytokines chemokine (C‐C motif) ligand 5 (CCL5) and IL‐8, and the precancer‐related factors CDX2 and MUC2 (Fig 1C) 37. The fusobacterium adhesion A (FadA) of *Fusobacterium nucleatum* (*F. nucleatum*) can bind the extracellular domain of E‐cadherin, thereby inducing phosphorylation and internalization of E‐cadherin. This then releases β‐catenin to activate β‐catenin–T‐cell factor (Tcf)/LEF, downstream in the Wnt signalling pathway to control transcription of genes involved in apoptosis, cell proliferation and transformation (Fig 1D) 38. In patients with colon adenomas or adenocarcinomas, high expression levels of *F. nucleatum* fadA have been associated with upregulated expression of oncogenic and inflammatory genes associated with the Wnt signalling pathway 39, 40.

The major surface‐exposed component of Gram‐negative bacteria, LPS additionally activates signalling cascades that promote cancer development. LPS is present in both pathogenic and commensal bacteria and plays a central role in the activation of TLR4. TLR4‐mediated signalling is critical for the downstream activation of numerous signalling pathways that underlie a variety of inflammatory and immune responses, and can promote the development of adenomatous polyposis coli (Apc)‐dependent colorectal cancers and inflammation‐associated colorectal cancers in mice. The role of TLR signalling in intestinal tumorigenesis has been studied through the crossing of myeloid differentiation primary response 88 (MyD88)‐deficient mice that have impaired TLR4 signalling, with *Apc* (*Apc* ^Min/*+*^) mice that mimic sporadic cancer and familial adenomatous polyposis. These MYD88‐deficient × *Apc* ^Min/*+*^ mice showed a reduction in both tumour number and size compared to the *Apc* ^Min/*+*^ control mice, suggesting that TLR4 signalling further promotes tumour growth 41, 42. Tumour tissues of mice lacking MyD88 showed lower expression of the Cox2 gene that is involved in inflammation, indicating a role of this gene in reduced tumour formation 43. It has furthermore been shown that Cox2 inhibitors, such as aspirin, reduce colorectal cancer risk in people that overexpress the 15‐PGDH gene which encodes for an enzyme that disrupts Cox2 activity 44. Studies with germ‐free and wild‐type mice showed that TLR4 activation by LPS from the intestinal microbiota pool contributes to the promotion of injury‐ and inflammation‐driven hepatocellular carcinoma by activating proliferative and anti‐apoptotic signals 19. Findings from these animal studies were further corroborated by human studies in which enhanced expression of the TLR4/MyD88 complex was detected in 20% of colorectal patient samples 45.

## Bacterial toxin‐mediated host cell transformation

To ensure immune escape, rapid replication and spreading, pathogenic bacteria do not only use immune‐evasion strategies to avoid host cell clearance, but are also capable of immune cell elimination. One of the strategies employed by bacteria is the secretion of protein toxins that have cytolytic properties. Bacteria can express protein toxins from their pathogenicity islands and secrete them through specialized secretion systems for transport across bacterial outer membranes 46. The interaction of proteins toxins with the host generally occurs in an ordered series of events and can be illustrated by the mode of action of the diphtheria toxin that inhibits the synthesis of host cell proteins through the inactivation of the host elongation factor 2 (EF‐2) protein. The diphtheria toxin consists of three subunits and is secreted by *Corynebacterium diphtheriae* as a single polypeptide chain. Diphtheria toxin then binds to the host's heparin‐binding epidermal growth factor‐like surface receptor that then is internalized in the endosomal system. Here, the transmembrane domain of the toxin is unfolded, which translocates the toxin to the cytosolic side of the endosomal membrane. This is followed by a reduction in the disulphide bond between toxin fragments A and B and release of the C‐domain into the cytoplasm. The C‐domain is then refolded into an enzymatically active conformation that catalyses NAD^+^‐dependent ADP‐ribosylation of EF‐2. This then inhibits protein synthesis, ultimately resulting in cell death of the targeted cells 47.

**Figure 2:**
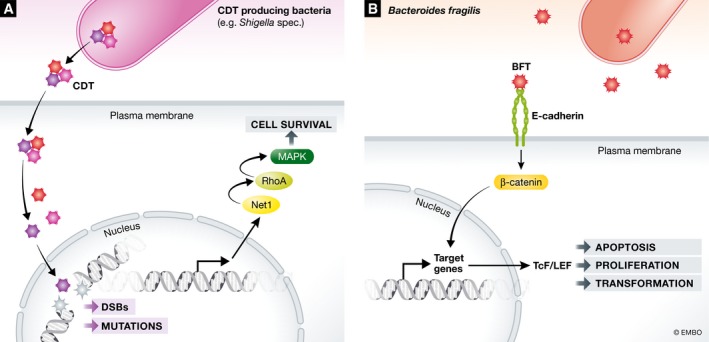
Host cell signalling pathways involved in cell growth and transformation manipulated by bacterial toxins (A) The CdtB subunit of the CDT toxin is delivered to the nucleus where it causes DSBs and impairs DNA DDR sensor functionality. At the same time, NET1 and RhoA are activated which ensure upregulation of MAPK and cellular survival. (B) *Bacteroides fragilis* BFT binds to E‐cadherin and involved in cellular signalling, proliferation and transformation via activation of the β‐catenin/Wnt and NF‐κβ signalling pathways.

Although pathogenic bacteria primarily use their toxin‐mediated assault strategies to create a favourable host cell environment, their toxins, likely as a side effect of their mode of action, can also contribute to carcinogenesis. Toxin‐mediated carcinogenesis can occur in multiple ways, including the induction of genomic instability, the induction of cell death resistance cell signalling and the induction of proliferative signalling 48. Genome instability is most readily caused by bacterial protein toxins that induce host cell double‐stranded DNA breaks, including the cytolethal distending toxin (CDT), the colibactin, the Shiga toxin and endonucleases. CDT is secreted by various Gram‐negative bacteria that belong to the Gamma and Epsilon class of Proteobacteria, including *S.* Typhi, *E. coli*,* Shigella dysenteriae* and *Campylobacter jejuni*. CDT is comprised of three subunits, CdtA, CdtB and CdtC. CdtA and CdtC ensure the uptake and cellular delivery of CdtB, which harbours the catalytic activity of CDT and causes double‐strand DNA breaks (DSBs) in host cells. After host cell binding and internalization by subunits CdtA and CdtC, CdtB undergoes retrograde transport via the endosomes and Golgi to the endoplasmic reticulum (ER), where it undergoes ER‐associated protein degradation‐mediated translocation into the cytosol. The CtdB subunit is then imported in the nucleus where it induces DSBs 49. These DSBs result in DNA damage responses (DDR) that cause G1‐S cell cycle arrest in endothelial and epithelial cells, and both G1‐S and G2‐M cell cycle arrest in fibroblasts and apoptosis in haematopoietic cells that are particularly sensitive to these toxins. As a result, this toxin can locally eliminate immune cells, providing an obvious advantage for the bacteria. However, prolonged exposure to sublethal doses of CDT can impair DDR sensor functionality, resulting in impaired detection of DNA damage and the accumulation of mutations. At the same time, mitogen‐activated protein kinase (MAPK) activity is upregulated by activation of the neuroepithelial cell‐transforming gene 1 protein (NET1) and the GTPase RhoA, which supports survival of the toxin‐exposed cells (Fig 2A) 50. As a consequence, these cells can propagate with DNA mutations and deletions that arise during the repair process, thus inducing genomic errors that underlie cancer formation.

In addition to the CDT toxins, the DNA interacting colibactin toxin has also been associated with the formation of DSBs and the introduction of genomic instability. Colibactin is secreted by *E. coli* strains of the phylogenetic group B2 that harbours the polyketide synthetase (pks) island 51. Bacteria that harbour the pks genomic island are able to induce DSBs in eukaryotic cells, which results in the activation of the DNA damage checkpoint pathways ATM, CHK1 and CHK2. This then results in CDC25 and cyclin‐dependent kinase 1 (CDK1)‐mediated G2‐ to M‐phase cell cycle arrest and finally in apoptotic cell death. As a side effect of their mode of action, colibactin‐producing bacteria also induce incomplete DNA repair, chromosomal instability and anchorage‐dependent colony formation, phenotypes that can promote cancer formation 52, 53. This is further substantiated by epidemiological studies showing that colibactin‐producing *E. coli* bacteria appear with high prevalence in biopsies of patients with human colorectal tumours 54, 55. Moreover, colitis‐susceptible IL‐10‐deficient mice showed increased formation of invasive carcinoma when colonized with *E. coli* secreting colibactin, whereas deletion of the pks genotoxic island from these *E. coli* strains decreased tumour multiplicity and invasion 56.

Besides toxins that contribute to carcinogenesis by introducing DSBs and genomic instability, toxins have been reported that promote carcinogenesis by inducing resistance to cell death signalling and by promoting proliferative signalling. These toxins are generally secreted by pathogenic bacteria that favour an intracellular host cell life as part of their infectious cycle and thus directly benefit from host cell survival. An example of such a toxin is the *Bacteroides fragilis* (*B. fragilis*) toxin (BFT) that binds to intestinal epithelial cell receptors and stimulates cell proliferation by cleavage of the tumour suppressor protein E‐cadherin 57, 58. E‐cadherin is involved in the formation of intercellular adhesion junctions in the intestinal epithelium and is involved in cellular signalling, proliferation and differentiation via activation of the β‐catenin/Wnt and NF‐κβ signalling pathways (Fig 2B) 59, 60, 61. BFT induced acute and chronic colitis in C57BL/six mice, and colon tumours in the multiple intestinal neoplasia (*Apc* ^Min/+^) mouse model for human colon carcinoma. This is the same mouse model where *H. pylori* triggers a pro‐carcinogenic multi‐step inflammatory cascade that requires IL‐17R, NF‐κβ and STAT3 signalling in colonic epithelial cells 62, 63. These mouse experiments are further substantiated by epidemiology, indicating that infections with enterotoxigenic variants of *B. fragilis*, as opposed to non‐toxigenic variants, are more prevalent in people with colorectal cancers. More specifically, the enterotoxigenic variant is present in only 10–20% of the healthy population, whereas 40% of CRC patients present enterotoxigenic *B. fragilis* in their faeces 64. In addition to BFT, multiple biologically plausible mechanisms have been reported that explain how the vacuolating cytotoxin A (VacA) of *H. pylori* enhances gastric cancer risk. Similar as the *H. pylori* outer membrane protein OipA, VacA activates the EGFR receptor that triggers PI3K–Akt signalling, and inactivates glycogen synthase kinase 3β 34, 65. As a result, β‐catenin degradation is abolished, which promotes Tcf/LEF‐controlled transcription that promotes cell growth and transformation 34, 65, 66. Another *H. pylori* virulence factor, cytotoxin‐associated gene A (CagA), which depends on the type IV pilus cell‐surface adhesion CagL for its host cell targeting, interacts with the c‐Met receptor to activate epithelial proliferation, as shown in human gastric organoids 67. Phosphorylated and unphosphorylated CagA can also interact with a variety of host proteins involved in the MEK, ERK, NF‐κβ and β‐catenin pathways that are all involved in host cell proliferation and cancer formation 68, 69.

## Bacterial effector proteins that mediate host cell transformation

Various intracellular bacterial pathogens have developed molecular mechanisms to ensure a persistent infection within the protective environment of the host cell's interior. This requires host cell control at various steps of the intracellular infection cycle, including host cell internalization through receptor‐mediated endocytosis or phagocytosis, intracellular survival and growth, and release from the infected host cell.

After host cell internalization bacterial‐cargo generally routes across the endosomal system that usually terminates in a highly degradative organelle, the phagolysosome. To avoid phagolysosomal degradation, intracellular bacterial pathogens have evolved various mechanisms that can be broadly grouped into pathways where pathogenic bacteria either escape the phagosome or enter in the cytosol, and pathways where the phagosome is hijacked and tailored to the preferences of the bacteria. Cytosolic pathogens like *Listeria*,* Shigella flexneri (S. flexneri)*,* Rickettsia* and *Francisella* are known to rapidly escape the phagosome to enter the host cytosol and thereby avoid lysosomal fusion and degradation 70. This generally involves secretion of bacterial effector proteins that induce pore formation of the endolysosomal vacuole and ensure its subsequent rupture. It has, for example, been shown that *S. flexneri* secretes the effector protein Invasion plasmid antigen B that forms ion channels in eukaryotic membranes and can mediate potassium influx and subsequent endolysosomal leakage 71. In addition, *Listeria* can secrete the listeriolysin‐O protein that induces small‐membrane perforations, which causes Ca^2+^ leakage from vacuoles and an increase in the vacuolar pH. Subsequently, vacuolar maturation is prevented 72, 73. *Francisella tularensis* (*F. tularensis*) also escapes into the host cytoplasm. After phagocytic uptake by macrophages, *F. tularensis* resides in the *Francisella‐*containing phagosome (FCP) that over time matures from a phagosome with an early endosomal character into a more acidic late endosomal phagosome. Since inhibition of FCP acidification delays the escape of *F. tularensis* into the cytosol, further acidification during phagosome maturation apparently stimulates *F. tularensis* to express unique, as‐yet‐undefined factors to disrupt the phagosomal membrane 74, 75, 76.

In contrast to bacteria that escape the phagosome, pathogenic bacteria have been reported that hijack the phagosome to ensure a favourable replication niche. An example of such a pathogen is *Legionella pneumophila* that redirects the *Legionella*‐containing phagosome to the ER via the secretion of bacterial proteins through the Dot‐Icm secretion system. This rearrangement prevents lysosomal degradation and ensures *Legionella* replication within the phagosome 77, 78. Bacterial control of phagosomal maturation has also been reported for the intracellular pathogen *Salmonella*. After its host cell internalization, *Salmonella* ends up in a membrane‐bound phagosome‐like vacuolar compartment called the *Salmonella*‐containing vacuole (SCV). The SCV then matures and acquires characteristics of late endocytic compartments including acidification. It does, however, not become bactericidal. Under control of the *Salmonella* effectors, SifA, SseJ, SseG, SseF, SopD2, and PipB2, cellular host processes are manipulated to turn the SCV into a compartment that facilitates *Salmonella* replication 79. SifA, which is critical in this process 80, interacts with the host cell effector of the GTPase Arl8b, the SifA and kinesin‐interacting protein SKIP. This interaction results in the formation of a tubular membrane network, known as *Salmonella*‐induced filaments, that is essential for the supply of nutrients to the SCV and prevents endosomal antimicrobial activities due to constant mixing of antimicrobial agents with late endosomes and lysosomes 81, 82.

**Figure 3:**
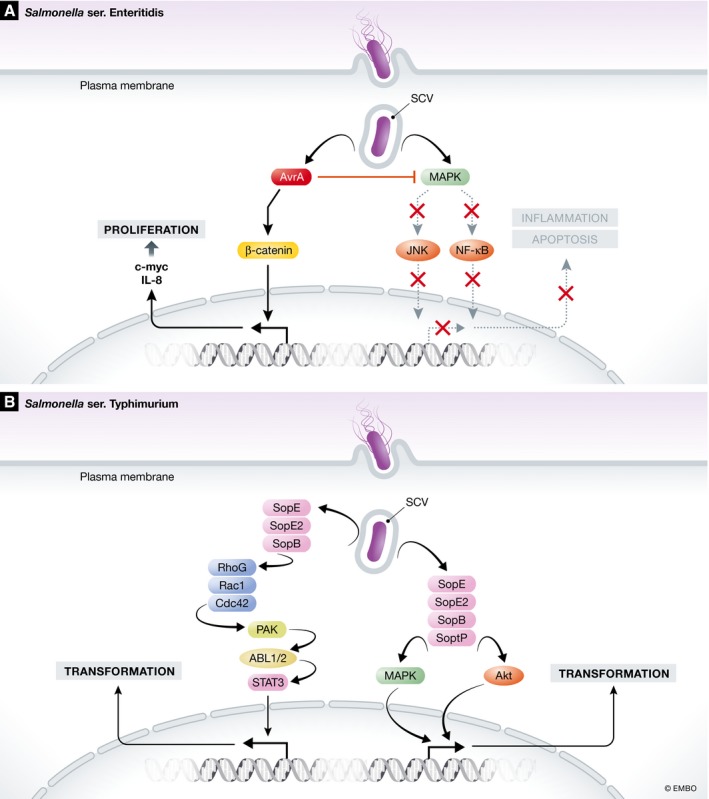
Examples of bacterial effector proteins involved in cellular transformation (A) *Salmonella* Enteritidis AvrA stabilizes β‐catenin, which results in proliferative Wnt signalling. At the same time, AvrA inhibits JNK and NF‐κβ signalling pathways involved in inflammation and apoptosis. (B) The effector proteins SopB, SopE and SopE2 of *Salmonella typhimurium* activate the small GTPases RhoG, Rac1 and Cdc42 and activate members of the PAK that phosphorylate members of the Abl kinase family, leading to the activation the cytoplasmic transcription factor STAT3, which contributes to cellular transformation. The effector proteins SopB, SopE, SopE2 and SptP of *S. typhimurium* additionally mediate activation of the MAPK and Akt pathways, which transforms premutated fibroblasts and gallbladder organoids.

Intracellular pathogenic bacteria that engage effector proteins during their intracellular life cycle manipulate host cell integrity in a major way. To this end, some of these infections have been epidemiologically linked to particular cancer types. Infections by two food‐borne *Salmonella* serovars, *S*. Typhi and *S*. Enteritidis, are linked to gallbladder carcinoma and colon cancer, respectively 13, 14. These bacteria introduce a series of effector proteins in the host cell to take over host cell biology and—depending on host pathway affected—can contribute to cancer formation. A *Salmonella* effector protein that has been linked to colon cancer formation is the acetyltransferase AvrA that alters a variety of host‐signalling pathways and modulates immune responses, apoptosis and proliferation 83, 84. AvrA modifies and stabilizes β‐catenin, thereby enhancing signalling and promoting epithelial cell proliferation (Fig 3A) 85, 86, 87. AvrA also suppresses the host immune system and its apoptotic defences via the inhibition of the c‐Jun N‐terminal kinase (JNK) and NF‐κβ signalling pathways (Fig 3A) 88. In addition to AvrA, three AvrA orthologues have been reported that similarly interact with essential host cell signalling pathways. However, in contrast to AvrA these orthologues have primarily only inhibitory effects on the host immune system. YopJ is expressed by *Yersinia pestis* and attenuates the ERK, p38, JNK and Iκβ kinase (IKK) pathways involved in the synthesis of cytokines as well as anti‐apoptotic factors 89. VopA of *Vibrio parahaemolyticus* can similarly inhibit host ERK, p38 and JNK signalling, but not the IKK pathway 90, 91, and AopP of *Aeromonas salmonicida* interacts with the IKK pathway 92.

In epithelial cells infected with *S*. *typhimurium*, the effector proteins SopE, SopE2 and SopB can manipulate host Rho‐family GTPases, p21‑activated kinase (PAK) and ABL tyrosine kinase to activate STAT3 and alter transcription regulation, which 93 can mediate transformation of cells (Fig 3B). In addition, cellular transformation can occur through *Salmonella* effector SopE, SopE2, SopB and SptP‐mediated activation of the MAPK and AKT pathways (Fig 3B) 94. The activation of these signalling pathways enables the transformation of fibroblasts and gallbladder organoids that harbour a pre‐transformed phenotype whereby the tumour suppressor gene p53 is inactivated and the MYC oncogene is overexpressed. These findings are supported by pathology on gallbladder carcinoma samples from Indian patients that contain both *S*. Typhi DNA and the pre‐transformed modifications also observed in the laboratory experiments, and by an *Apc* ^Min/+^ mouse model in which oral infection with *S*. *typhimurium* results in the development colorectal adenocarcinomas in a *Salmonella* effector‐dependent manner 13.

## Conclusions

Although bacterially induced host cell manipulation can promote cancer formation, it is unlikely that bacterial pathogens themselves experience any evolutionary benefit from their carcinogenic actions. Bacterially induced cancer formation is more likely an unfortunate consequence of the bacterial infection cycle since cancer usually occurs long after the bacterium and its effectors have left the host 13, 14. Moreover, bacterial host cell manipulations involved in the induction of cancer formation usually account for only one step in the multi‐step process required for actual cellular transformation and cancer formation. This can be illustrated by *Salmonella* infections that only in combination with pre‐mutations allow cellular transformation in tissue culture fibroblasts and gallbladder organoids and is supported by observations of Indian gallbladder cancer patients who showed the corresponding pre‐mutations in the p53 gene, c‐MYC amplification in their tumours and had a history of *S*. Typhi infection. 11 In other words, *Salmonella* will only induce cancer when the cell has made already one or multiple pretransforming steps. This would explain why chronic bacterial infections have a higher statistical chance of initiating tumorigenesis as the likelihood of encountering a pre‐transformed cell would then be markedly increased. This may also explain the correlations of persistent *Mycobacterium tuberculosis* infections and pulmonary cancers 95 and chronic *Coxiella burnetii* infections and B‐cell non‐Hodgkin lymphoma 96.

Since many epidemiological studies reveal a link between bacterial infections and cancer incidence, and the number of bacterial mechanisms that can contribute to cellular transformation are most likely considerable larger than reported to date, we expect that the number of examples illustrating the role of bacterial infections in cancer formation will increase the coming years. It is also known that bacterial effectors from different species can act synergistically during host cell manipulation and then act in a symbiotic interspecies manner 55, 97. These combined mechanisms can induce cell transformation and cancer in an even more complex manner and further contribute to the complexity of bacterial contributions to cancer.

While the first examples of bacterial mechanisms contributing to cancer are uncovered, it is likely that bacteria will provide many new and surprising mechanisms for host cell manipulation, some of which may participate in cell transformation. These may include an expansion of mechanisms involved in immune evasion, DNA damage and signalling pathways, but may also include more indirect routes, as, for example, via the formation of carcinogenic metabolites 98. When the role of defined bacterial mechanisms in cancer formation will become more apparent and accepted (see also Box 1), studies on their prevention or control can help reduce cancer formation. On this note, antibiotic therapy during cancer treatment, which is already a standard of care in patients with gastric mucosa‐associated lymphoid tissue (MALT) lymphoma 99, might become a valuable addition to current tumour‐targeting therapies. This, however, may only help when the presence of a bacterial species is required to continuously provide signals to maintain the transformed state. Otherwise, patients diagnosed with a bacterial pathogen known to participate in cancer formation—but not necessarily maintenance—may be incorporated in cancer screening programs.

**Box 1: In need of answers**

- Do bacterial infections only decrease the threshold for cellular transformation or can they also initiate tumour formation?
- How is transformation by activation of host signalling pathways imprinted in host cells?
- How can correlations from microbiome studies be translated to causalities?
- Does transformed tissue cause microbial dysbiosis 100?
- It has been shown that there is a distal oncogenic effect of the gut microbiome 101. How does the gut microbiome affect tumour formation at a distance?
- What is the total contribution of bacteria to cancer formation?
- How to translate the collective knowledge on bacteria and cancer formation into treatment or prevention measures?

## Conflict of interest

The authors declare that they have no conflict of interest.
